# Multiple MoS_2_ Transistors for Sensing Molecule Interaction Kinetics

**DOI:** 10.1038/srep10546

**Published:** 2015-05-27

**Authors:** Hongsuk Nam, Bo-Ram Oh, Pengyu Chen, Mikai Chen, Sungjin Wi, Wenjie Wan, Katsuo Kurabayashi, Xiaogan Liang

**Affiliations:** 1 Department of Mechanical Engineering, University of Michigan, Ann Arbor, MI 48109; 2 University of Michigan-Shanghai Jiao Tong University Joint Institute and Department of Physics and Astronomy, Shanghai Jiao Tong University, Shanghai 200240, China

## Abstract

Atomically layered transition metal dichalcogenides (TMDCs) exhibit a significant potential to enable next-generation low-cost transistor biosensors that permit single-molecule-level quantification of biomolecules. To realize such potential biosensing capability, device-oriented research is needed for calibrating the sensor responses to enable the quantification of the affinities/kinetics of biomolecule interactions. In this work, we demonstrated MoS_2_-based transistor biosensors capable of detecting tumor necrosis factor – alpha (TNF-**α**) with a detection limit as low as 60 fM. Such a detection limit was achieved in both linear and subthreshold regimes of MoS_2_ transistors. In both regimes, all sets of transistors exhibited consistent calibrated responses with respect to TNF-**α** concentration, and they resulted in a standard curve, from which the equilibrium constant of the antibody-(TNF-**α**) pair was extracted to be K_D_ = 369 ± 48 fM. Based on this calibrated sensor model, the time-dependent binding kinetics was also measured and the association/dissociation rates of the antibody-(TNF-**α**) pair were extracted to be (5.03 ± 0.16) × 10^8^ M^−1^s^−1^ and (1.97 ± 0.08) × 10^−4^ s^−1^, respectively. This work advanced the critical device physics for leveraging the excellent electronic/structural properties of TMDCs in biosensing applications as well as the research capability in analyzing the biomolecule interactions with fM-level sensitivities.

Using field-effect transistor (FET)-based biosensors created from nanowires (NWs) and carbon nanotubes (CNTs), researchers have demonstrated detection of cancer biomarkers from nM to fM range in serum^1^,^2^,^3^,^4^,^5^,^6^,^7^, *in vitro* detection of nM proteins in cell growth systems^8^,^9^, and quantification of the affinities/kinetics of the protein interactions with fM-level sensitivities^10^. The fM-level limit-of-detection (LOD) achieved by such nanoscale FET biosensors for monitoring biomarker concentrations would enable label-free, single-molecule-level detection of trace-level amount biomarkers. The arrays of such biosensors with consistent transistor responses would serve as reliable lab-on-a-chip platforms for precisely determining the kinetics of various biomolecule interactions. However, serious constraints imposed on nanofabrication severely prohibit the reliable manufacturing of the affordable biosensor chips utilizing such one dimensional (1D) nanostructures^1^,^5^,^6^. In particular, high-quality, small-size NWs and CNTs are needed to make biosensors with fM-level LOD for concentration monitoring (or single-molecule-level LOD for trace-level amount detection)^11^. Especially, for trace-level amount detection, the critical dimensions of the sensing channels need to be comparable to the impact dimensions of charged molecules to maximize the gating effect due to the charged molecules and achieve very low LOD^4^,^12^,^13^. CNTs and many NWs are usually produced by using bottom-up synthesis methods (*e.g.*, chemical vapor deposition (CVD)). The community currently lacks proper top-down planar nanofabrication processes to produce ordered arrays of such nanostructures, which makes it very challenging to realize parallel high-throughput assay using biosensors made from these nanostructures. High-quality Si NW biosensor arrays can be made using top-down lithographic techniques^10^. However, the fabrication of such Si NW arrays usually needs expensive semiconductor-on-insulator (SOI) substrates and exquisite nanolithographic tools, which can result in a high processing cost and is not very suitable for manufacturing affordable (even disposable) assay chips for practical clinical biosensing applications.

Emerging two-dimensional (2D) atomically layered materials, such as graphene, topological insulators (TIs), and TMDCs, recently attracted a great deal of interest because of their attractive electronic/optoelectronic properties, large abundance, and compatibility to planar nanofabrication processes^14^,^15^,^16^,^17^,^18^,^19^,^20^,^21^,^22^,^23^,^24^,^25^. Due to their atomically thin structures, the transport properties of 2D layers are highly sensitive to the external stimuli, which can enable new ultrasensitive 2D FETs suitable for biosensing applications^26^,^27^,^28^,^29^,^30^. Especially, in comparison with the thin film transistors made from conventional bulk semiconductors (*e.g.*, Si and III-V compounds), the transistors based on MoS_2_ and other atomically layered semiconductors are expected to exhibit much more sensitive electrical responses to antigen-antibody binding events. Furthermore, all 2D layers have an extremely low density of dangling bonds on their surfaces, which can result in high-quality FET channels with low densities of scattering centers (and hence low Flicker noise level), and enable highly sensitive, low-noise-level detecting of biomolecules^27^,^31^,^32^,^33^,^34^. Novoselov *et al.* have demonstrated graphene-based FET sensors capable of detecting individual gas molecules absorbed on the graphene channels^35^,^36^.

In contrast to zero-bandgap graphene, semiconducting TMDCs (*e.g.*, MoS_2_) have sizable bandgaps. Therefore, TMDC-based FETs exhibit high On/Off current ratios up to 10^8^, which, in combination with their atomically thin structures, can potentially enable the higher detection sensitivities for gas, chemical, and biological sensing applications in comparison with graphene FETs^37^,^38^,^39^. Wang *et al.* and Sarkar *et al.* recently demonstrated that FET biosensors made from microscale few-layer-MoS_2_ flakes exhibit 100–400 fM LODs for detecting cancer-related biomarkers^39^,^40^. These previous works strongly imply that such TMDC-based FET biosensors may not need sensing channels of nanoscale width to achieve fM-level LODs for concentration monitoring applications, and the fabrication of such biosensors would not need exquisite nanolithographic tools. In addition, several recent nanomanufacturing-related works suggest that monolayer/few-layer TMDC structures and other relevant atomically layered materials hold significant potential to be produced over large areas on low-cost substrates (*e.g.*, glass, plastic, or rubber) by using cost-efficient processes such as CVD followed with roll-to-roll transfer^41^, addressable deposition^42^, and microscale stamping^43^,^44^,^45^. Therefore, it is very promising to realize cost-efficient manufacturing of multiplexing assays based on TMDC transistor arrays in the future.

Toward such envisaged bio-assay capability, additional device-oriented research is needed for quantitatively calibrating the sensor responses measured from multiple sets of TMDC FET biosensors, so that the calibrated response signals are consistent with each other and can synergistically enable precise quantification of biomarker concentrations (or amounts) as well as the affinities/kinetics of biomolecule interactions. Although individual MoS_2_ FET biosensors have been fabricated and exhibited very high biodetection sensitivity^39^,^40^. the utilization of multiple such devices for quantifying the biomolecule interactions has not been attempted.

In this work, we fabricated multiple sets of MoS_2_-based transistor biosensors and demonstrated that these devices can be synergistically utilized to measure the concentrations of analyte solutions as well as the affinity and kinetic properties of the analyte-receptor pair. The biomolecule under study, TNF-α, is a pro-inflammatory cytokine and a key biomarker associated with host defense and immunosurveillance^46^,^47^,^48^,^49^. Researchers have shown that TNF-α secreted from immune cells stimulated with lipopolysaccharide (LPS) – an endotoxin causing septic shock due to severely pronounced immune response of the human body – reflects a functioning innate immune response^50^,^51^. All our biosensors exhibited a TNF-α detection limit as low as 60 fM despite the small molecular size of the cytokine biomarker (~17 kDa) that renders its label-free detection at high sensitivity significantly challenging. Such a low detection limit was achieved in both linear and subthreshold regimes of the transfer characteristics of MoS_2_ transistors. In both transport regimes, the electrically measured sensor responses were calibrated into signal quantities independent of the transistor performance. All sets of transistor biosensors exhibited consistent relationships between calibrated sensor responses and TNF-α concentrations. They generated a standard curve, from which the equilibrium constant of the antibody-(TNF-α) pair was extracted to be K_D_ = 369 ± 48 fM. Based on this calibrated sensor model, the time-dependent association-dissociation kinetics of the antibody-(TNF-α) pair was further studied and the association/dissociation rates of the antibody-(TNF-α) pair were measured to be (5.03 ± 0.16) × 10^8^ M^−1^s^−1^ and (1.97 ± 0.08) × 10^−4^ s^−1^, respectively.

## Results and Discussion

**Figure 1:**
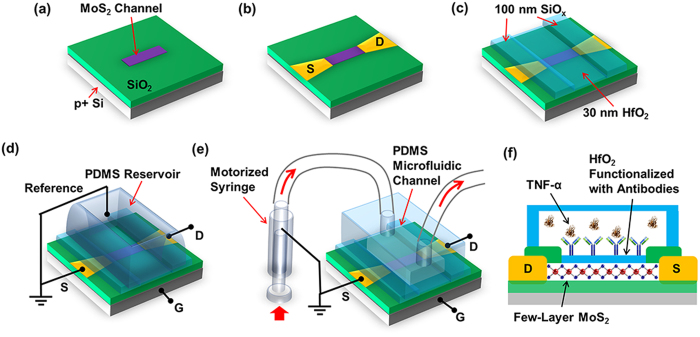
Flow chart for fabricating a MoS_2_ transistor biosensor: (**a**) printing of a few-layer MoS_2_ flake onto a p^+^-Si/SiO_2_ substrate; (**b**) fabrication of Ti/Au D/S contacts; (**c**) ALD growth of the HfO_2_ effective layer on top of the MoS_2_ channel and coating of D/S contacts with thick SiO_x_ layers; (**d**) integration of a PDMS liquid reservoir on top of the MoS_2_ transistor for measuring sensor responses from different TNF-α concentrations under thermodynamic equilibrium condition and determining the affinity of the antibody-(TNF-α) pair; (**e**) integration of a microfluidic inlet/outlet tubing kit driven by a motorized syringe pump on top of the transistor for quantifying the association-dissociation kinetics of the antibody-(TNF-α) pair; (**f**) functionalization of the HfO_2_ effective layer with antibody receptors and subsequent TNF-α detection.

Figure 1 illustrates the steps for fabricating a transistor biosensor with a few-layer MoS_2_ sensing channel. First, a pristine few-layer MoS_2_ flake is printed onto a p^+^-doped Si substrate coated with 300 nm thick SiO_2_ (Fig. 1(a)). This printing process is the same as the method previously reported by us^45^. The thickness of the MoS_2_ flake chosen for making a biosensor is specifically controlled to be 15–20 nm, aiming to achieve relatively high field-effect mobility values (*μ* = 20 to 30 cm^2^/Vs)^52^,^53^. After the MoS_2_ printing, metallic drain/source (D/S) contacts (5 nm Ti/50 nm Au) are fabricated using photolithography followed with metal deposition and lift-off, and a back-gated MoS_2_ transistor is subsequently formed (Fig. 1(b)). To enable a capacitive coupling between the microfluidic reservoir (or channel) and the MoS_2_ transistor channel, a 30 nm thick HfO_2_ layer is deposited on top of the MoS_2_ channel using atomic layer deposition (ALD) (Fig. 1(c)). This HfO_2_ layer also serves as an effective layer for biofunctionalization. Afterwards, additional 100 nm thick SiO_x_ is sputtered on D/S contacts to minimize the leakage current between D/S contacts and microfluidic components (Fig. 1(c)). Before the TNF-α detection, anti-human TNF-α antibody is functionalized on the HfO_2_ effective layer. The detailed antibody functionalization procedure is illustrated in Fig. S1 in the supporting information and also described in the Method and Material section. To measure the MoS_2_ transistor sensor responses from different TNF-α concentrations under the thermodynamic equilibrium condition and determine the affinity of the antibody-(TNF-α) pair, a large open liquid reservoir made from polydimethylsiloxane (PDMS) is integrated on top of the MoS_2_ transistor (Fig. 1(d)). Such a setup is very simple and enables the quick loading of various analyte solutions. To measure the association-dissociation kinetics of the antibody-(TNF-α) pair, a microfluidic channel is integrated on top of the transistor sensor, and a motorized syringe pump is used for driving the TNF-α solution flow into and out of the microfluidic channel through an inlet/outlet tubing kit (Fig. 1(e)). Such a setup can enable stable laminar flows of analyte solutions and minimize the noise induced by the liquid loading processes, which is required for precisely analyzing the real-time kinetic processes of antibody-(TNF-α) binding. Figures 1(d) and (e) also illustrate the circuit setups for measuring the transistor sensor responses. In addition, Fig. 1(f) illustrates the cross-sectional view of a MoS_2_ transistor sensor in the TNF-α detection operation. Other device fabrication and characterization details are described in the Method and Material section.

**Figure 2:**
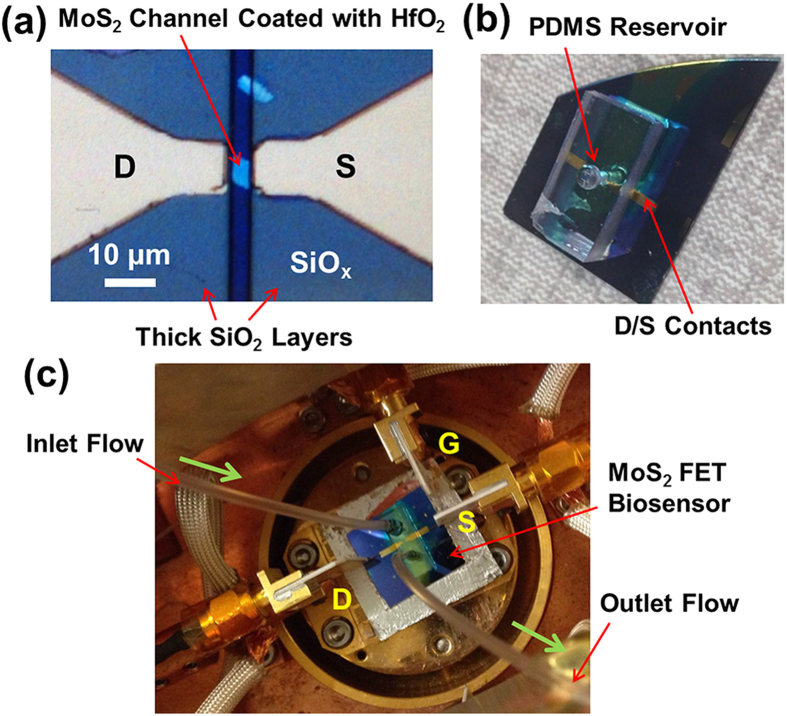
Optical micrographs or photographs of (**a**) an exemplary MoS_2_ transistor with channel length (*L*) and width (*W*) of 5 and 6 μm, respectively; (**b**) an as-fabricated MoS_2_ transistor biosensor integrated with a cylindrical liquid reservoir, which is drilled into a PDMS block and is ~4 mm deep and ~1 mm in diameter; (**c**) a transistor biosensor integrated with a microfluidic channel system connected with an inlet/outlet tubing kit, which is driven by a motorized syringe pump.

Figure 2(a) displays the optical micrograph (OM) of an exemplary MoS_2_ transistor with channel length (*L*) and width (*W*) of 5 and 6 μm, respectively. Figure 2(b) shows the photograph of an as-fabricated MoS_2_ transistor biosensor integrated with a PDMS liquid reservoir. The reservoir is ~4 mm deep and is ~1 mm in diameter, which is drilled into a PDMS block with length, width, thickness of 2, 1, and 0.4 cm, respectively. Figure 2(c) shows the photograph of a biosensor integrated with a microfluidic channel connected with an inlet/outlet tubing kit. Other details about the dimensions of PDMS fluidic components are described in the Method and Material section.

First we measured the sensor responses from different TNF-α concentrations under the thermodynamic equilibrium condition. The biosensor setup shown in Fig. 2(b) was used for this measurement. For each transistor biosensor, the static transfer characteristics (*i.e.*, drain-source current (*I*_*DS*_) – back gate voltage (*V*_*G*_) curves acquired under a fixed drain-source voltage (*V*_*DS*_)) were measured at each of the biodetection stages, following the sequence of (1) bare transistor, (2) antibody functionalization, and inputs of TNF-α solutions with concentrations of (3) 60 fM, (4) 300 fM, (5) 600 fM, (6) 3 pM, and (7) 6 pM. To eliminate the effect of the *I*_*DS*_*-V*_*G*_ hysteresis, all *I*_*DS*_*-V*_*G*_ curves were measured by sweeping *V*_*G*_ from -100 V to 100 V with a sweep rate of 10 V/s. Other details about different biodetection stages and transistor characterizations are described in the Method and Material section.

**Figure 3:**
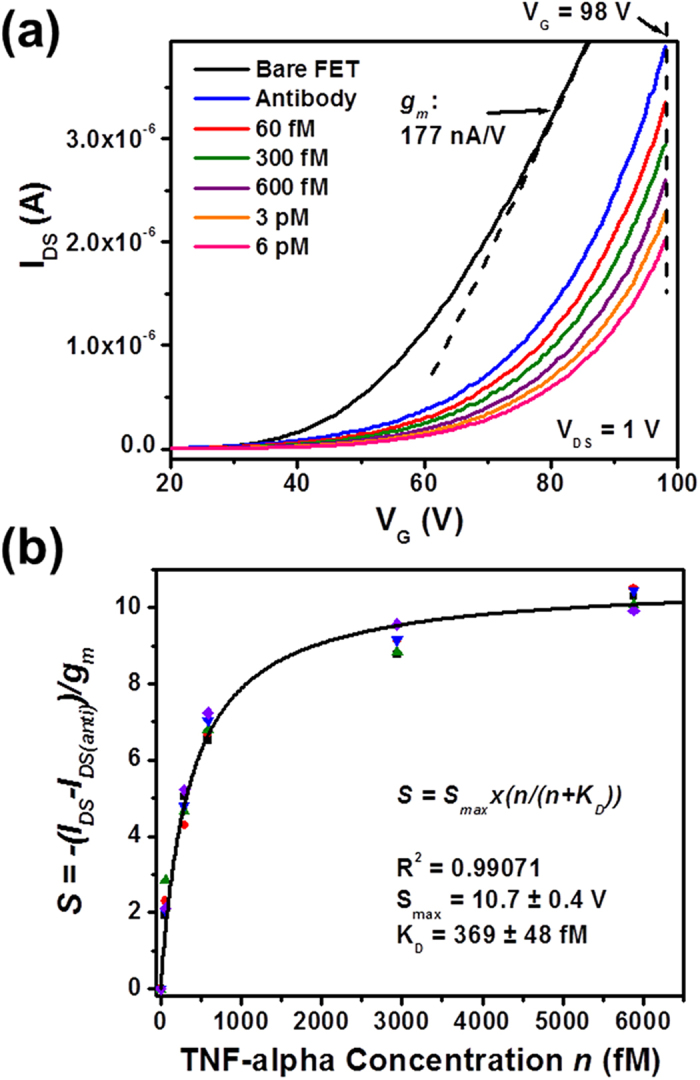
Sensor responses measured in the linear transport regimes of MoS_2_ transistor biosensors: (**a**) transfer characteristics of an exemplary MoS_2_ transistor sensor measured at various biodetection stages, following the sequence of (1) bare transistor, (2) antibody functionalization, and inputs of TNF-α solutions with concentrations of (3) 60 fM, (4) 300 fM, (5) 600 fM, (6) 3 pM, and (7) 6 pM; (**b**) a set of calibrated linear-regime responses (*S*) measured from five different MoS_2_ transistor sensors with respect to TNF-α concentration (*n*). These *S-n* relationships can be well fitted with Langmuir isotherms and the dissociation constant (K_D_) of the antibody-(TNF-α) pair is extracted to be 369 ± 48 fM.

Figure 3 displays the sensor responses measured in the linear transport regimes of MoS_2_ transistor sensors. Specifically, Fig. 3(a) shows the transfer characteristics of an exemplary sensor measured at various biodetection stages. Here, *I*_*DS*_ data are plotted in the linear scale. The transfer characteristics of this sensor exhibit a strong dependence on TNF-α concentrations, and the TNF-α detection limit is estimated to be ~60 fM. We choose a fixed *V*_*G*_ within the linear regimes of all *I*_*DS*_*-V*_*G*_ curves (*e.g.*, *V*_*G*_ = 98 V, as denoted by the dashed vertical line in Fig. 3(a)). The *I*_*DS*_ values measured under this *V*_*G*_ vary according to different biodetection states and such *I*_*DS*_ data could be utilized as a sensor response signal. However, such a response signal is highly dependent on the transistor performance parameters (*e.g.*, transconductance (*g*_*m*_) and threshold voltage (*V*_*T*_)). Therefore, in the analysis of a given biodetection state, the *I*_*DS*_ signals acquired by different MoS_2_ transistors may exhibit a poor device-to-device consistency due to the nonuniformity of MoS_2_ transistors. Although such an issue could be mitigated through optimizing the material deposition and device fabrication processes, a calibrated sensor response quantity independent of the device performance is highly desirable.

The linear regime of an *I*_*DS*_*-V*_*G*_ characteristic curve measured from a microscale MoS_2_ transistor sensor in a specific biodetection state can be expressed as Equation (1). In our experiments, it is observed that for a given transistor sensor, the *g*_*m*_ values extracted from different *I*_*DS*_*-V*_*G*_ curves that correspond to different biodetection states are very close and can be approximated as a constant for this sensor. For example, the *g*_*m*_ value of the sensor shown in Fig. 3(a) is extracted to be ~177 nS at *V*_*DS*_ = 1 V. Based on this observation and Equation (1) as well as the implication from previous works done by Duan *et al.* and Ishikaw *et al.*^10^,^54^, a calibrated sensor response quantity (*S*) is derived and expressed in Equation (2), where *I*_*DS(anti)*_ is the *I*_*DS*_ value measured in the “antibody functionalization” state of a sensor biased under a set of fixed *V*_*DS*_ and *V*_*G*_, and *I*_*DS*_*-I*_*DS(anti)*_ indicates the *I*_*DS*_ variation induced by the introduction of TNF-α molecules. Such an *I*_*DS*_ variation normalized by the *g*_*m*_ of this sensor results in a sensor response quantity directly related to the change in the *V*_*T*_ of the sensor (*i.e.*, *ΔV*_*T*_). It should be noted that although *ΔV*_*T*_ is assumed to be completely induced by the charge brought to the HfO_2_ effective layer on top of the transistor channel through antibody-(TNF-α) binding events, *ΔV*_*T*_ is not exactly the binding-event-induced potential change (*ΔΦ*) on the effective layer. This is because in this work, *ΔV*_*T*_ is the change in the *V*_*T*_ measured from the back gate. However, *ΔV*_*T*_ and *ΔΦ* can be related by *ΔV*_*T*_ *=* *(C*_*HfO2*_*/C*_*SiO2*_*)ΔΦ*, where *C*_*SiO2*_ and *C*_*HfO2*_ are the capacitances of the SiO_2_ back gate dielectric and the HfO_2_ effective layer, respectively. More detailed discussion about these potential parameters can be found from the dual-gate transistor model illustrated in Fig. S2. Based on this model, *ΔV*_*T*_ can be evaluated using 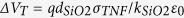, where *q* is the effective charge carried by a TNF-α molecule (the screening effect due to the buffer liquid has been incorporated into *q*); *d*_*SiO2*_ and *k*_*SiO2*_ are the thickness and dielectric constant of the SiO_2_ back-gate dielectric layer, respectively; *ε*_*0*_ is the vacuum permittivity; and *σ*_*TNF*_ is the areal density of TNF-α molecules bound to the antibody receptors functionalized on the effective layer. Therefore, such a calibrated response quantity (*S*) is proportional to the antibody receptor occupancy at the equilibrium state and it is also independent of the MoS_2_ transistor performance. These two conditions are critical for the subsequent Langmuir isotherm analysis.
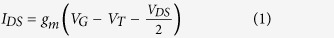

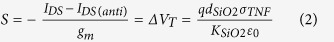


Figure 3(b) plots the calibrated responses measured from the linear transport regimes of five different sensors with respect to TNF-α concentration (*n*). The detailed transfer characteristics of these five devices measured at various biodetection stages are displayed in Fig. S3. Although Fig. S3 shows that the transfer characteristics of these five sensors exhibit significant difference in *V*_*T*_, *I*_*DS*_ and *g*_*m*_, Fig. 3(b) shows that the calibrated responses from these sensors are consistent with each other and can serve as a standard curve (*i.e.*, a generic *S-n* curve) for TNF-α detection. This standard curve can be well fitted with Langmuir isotherms (Equation (3)) and the affinity equilibrium (or dissociation) constant (K_D_) of the antibody-(TNF-α) pair is extracted to be 369 ± 48 fM; the maximum sensor response (*S*_*max*_) is extracted to be 10.7 ± 0.4 V.
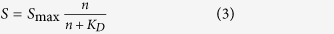


**Figure 4:**
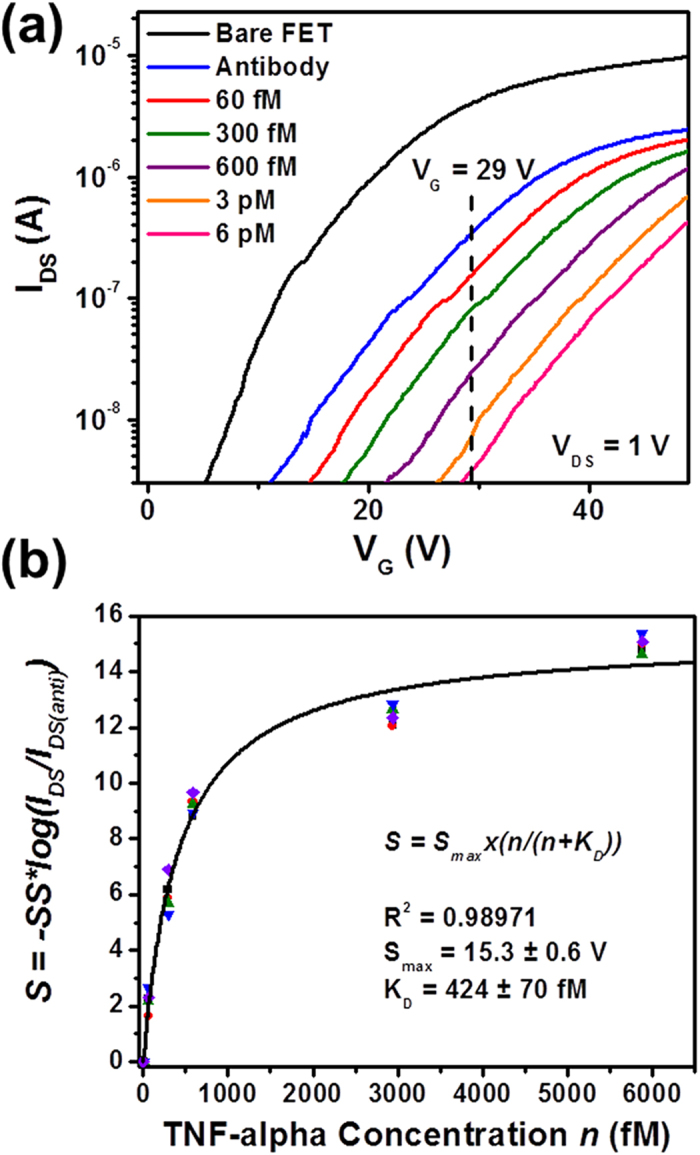
Sensor responses measured in the subthreshold regimes of MoS_2_ transistor biosensors: (a) transfer characteristics of an exemplary MoS_2_ transistor sensor measured at various biodetection stages, following the sequence of (1) bare transistor, (2) antibody functionalization, and inputs of TNF-α solutions with concentrations of (3) 60 fM, (4) 300 fM, (5) 600 fM, (6) 3 pM, and (7) 6 pM (Here *I*_*DS*_ data are plotted in the logarithm scale, and the subthreshold regimes are emphasized); (b) a set of calibrated subthreshold-regime responses (*S*) measured from five different MoS_2_ transistor sensors with respect to TNF-α concentration (*n*). These *S-n* relationships can be well fitted with Langmuir isotherms and the dissociation constant (K_D_) of the antibody-(TNF-α) pair is extracted to be 424 ± 70 fM.

Alternatively, sensor responses can also be measured from the subthreshold regimes of MoS_2_ transistor sensors. In the subthreshold regime of a transistor sensor, the sensitivity of *I*_*DS*_ to the variation of electrical potential (or charge) at the effective layer is much higher than that in the linear transport regime of this sensor. Therefore, the responses from the subthreshold regimes of transistor sensors are expected to result in the higher biodetection sensitivity in comparison with those from the linear regimes. Figure 4(a) shows the transfer characteristics of another exemplary MoS_2_ transistor sensor, which were measured at various biodetection stages. Here *I*_*DS*_ data are plotted in the logarithm scale, and the subthreshold regimes are emphasized. We choose a fixed *V*_*G*_ within the subthreshold regimes of all *I*_*DS*_*-V*_*G*_ curves (*e.g.*, *V*_*G*_ = 29 V denoted by the vertical dashed line in Fig. 4(a)). The *I*_*DS*_ values measured under this *V*_*G*_ clearly vary according to different biodetection states and exhibit a strong dependence on TNF-α concentration. Here, the TNF-α detection limit is estimated to be at least as low as 60 fM. Similarly, such *I*_*DS*_ data acquired in the subthreshold regimes of transistor sensors cannot be directly used as standard sensor responses. A calibrated subthreshold-regime response quantity independent of the transistor performance is needed.

In the subthreshold regime of a microscale MoS_2_ transistor sensor, the *I*_*DS*_*-V*_*G*_ relationship measured from a specific biodetection state can be approximately expressed by Equation (4), where *I*_*T*_ is the *I*_*DS*_ value measured at *V*_*G*_*=V*_*T*_ under a given *V*_*DS*_; *SS* is the subthreshold swing. As observed in our experiments, although the functionalization of a transistor sensor with antibody receptors (*i.e.*, the transition from “bare transistor” to “antibody functionalization” states) can result in an observable reduction of the *SS* of this sensor, the *SS* value does not significantly vary among the subsequent biodetection states, including the inputs of TNF-α samples with incremental concentrations. Therefore, for a given as-functionalized transistor sensor, *SS* can be approximated as a constant. Based on this observation, a calibrated subthreshold-regime sensor response quantity (*S*) is derived from Equation (4) and expressed in Equation (5), in which *I*_*DS(anti)*_ is the drain-source current measured in the “antibody functionalization” state of a sensor biased under a set of fixed *V*_*DS*_ and *V*_*G*_; and *I*_*DS*_ is the drain-source current measured from a subsequent biodetection state (*i.e.*, a specific TNF-α concentration). Similar to the calibrated linear-regime response quantity expressed in Equation (2), this subthreshold counterpart is also directly related to *ΔV*_*T*_, independent of the transistor performance, and proportional to σ_TNF_.
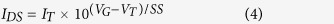

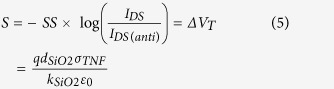


Figure 4(b) displays the calibrated subthreshold-regime responses (*S*) measured from five different sensors with respect to TNF-α concentration (*n*). The detailed transfer characteristics of these five devices measured at various biodetection stages are displayed in Fig. S4. As shown in Fig. S4, the transfer characteristics of these five sensors exhibits significant difference in *V*_*T*_, *I*_*DS*_ and *SS* parameters. However, as shown in Fig. 4(b), the calibrated *S-n* curves measured from these devices are consistent with each other and can be well fitted with Langmuir isotherms (Equation (3)). Here, the equilibrium constant (K_D_) of the antibody-(TNF-α) pair is extracted to be 424 ± 70 fM, which is consistent with the K_D_ value extracted from the linear-regime sensor responses (*i.e.*, 369 ± 48 fM). The *S*_*max*_ parameter is fitted to be 15.3 ± 0.6 V, which is about 40% larger than that extracted from the linear-regime responses (*i.e*., 10.7 ± 0.4 V). This observable discrepancy has not been fully understood. However, this could be temporarily attributed to the different back-gate *V*_*G*_ levels required for biasing sensors in subthreshold and linear regimes, which could result in different magnitudes of electric field penetrating through few-layer MoS_2_ channels as well as HfO_2_ effective layers and leaking into the analyte solution. This could lead to different degrees of the modification of electrical-double-layers around sensors and therefore different degrees of the screening of the charges brought through analyte-receptor binding pairs.

**Figure 5:**
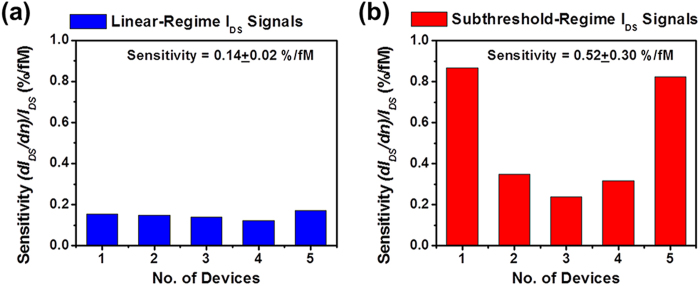
Sensitivity data acquired from (**a**) the linear-regime *I*_*DS*_ signals measured from the five sensors shown in Fig. S3 and (**b**) the subthreshold-regime *I*_*DS*_ signals measured from the five sensors shown in Fig. S4. All differential sensitivities were evaluated at TNF-α concentration of *n* = 60 fM (*i.e.*, 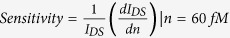).

Although the *I*_*DS*_ signals measured from both linear and subthreshold regimes can be mathematically normalized to consistent device-independent response quantities using Equations (2) and (5), the physical limit-of-detection of a transistor biosensor is indeed determined by the sensitivity of *I*_*DS*_ to the variation of analyte concentration (*dn*) as well as the noise level of electrically measured *I*_*DS*_ signals. This *I*_*DS*_ sensitivity is quantitatively defined as the relative change in *I*_*DS*_ per change in *n* (*i.e.*, 
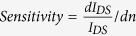
). Figure 5 displays and compares the sensitivity data acquired from (a) the linear-regime *I*_*DS*_ signals measured from the five sensors shown in Fig. S3 and (b) the subthreshold-regime *I*_*DS*_ signals from the five sensors shown in Fig. S4. All differential sensitivity values are evaluated at TNF-α concentration of *n* = 60 fM. This can provide critical information about the sensitivity required for obtaining fM-level detection limits. Figure 5 shows that the subthreshold-regime *I*_*DS*_ sensitivities (0.52 ± 0.3%/fM) are statistically higher than the linear-regime *I*_*DS*_ sensitivities (0.14 ± 0.02%/fM). Therefore, subthreshold-regime sensor responses are more desirable in achieving high detection sensitivity. However, it should be noted that the ultimate detection limit of a transistor sensor is also limited by the signal-to-noise ratios of electrically measured *I*_*DS*_ signals. In addition, for detecting low-abundance molecules, the non-specific adsorption of target molecules could also strongly affect the detection limit. The further analysis of these aspects is beyond the scope of this work but will be addressed in the future research. Finally, it is also noted that the sensitivity data listed in Fig. 5(b) exhibit the larger device-to-device variation in comparison with those listed in Fig. 5(a). This is probably because of that the subthreshold swing properties of MoS_2_ transistors are more sensitive to the fabrication-introduced defects than their linear-regime transconductance properties. Therefore, for our current MoS_2_ transistors, their linear-regime I_DS_ signals (or linear-regime transconductances) exhibit the higher device-to-device consistency than their subthreshold-regime I_DS_ signals.

To evaluate the detection specificity of our MoS_2_ transistor sensors, a sensor functionalized with anti-human TNF-α antibody is used for detecting interleukin-6 (IL-6) cytokine. Figure S5 shows the transfer characteristics of this sensor measured at various stages, including (1) bare transistor, (2) antibody functionalization, and inputs of IL-6 solutions with concentrations of (3) 600 fM and (4) 6 pM. Figure S5 shows that the presence of IL-6 that is not specific to TNF-α antibody cannot result in prominent change in the transfer characteristics. Such experimentally observed weak sensor responses to IL-6 indicate a negligible nonspecific adsorption of IL-6 molecules on the sensor surface, which may be effectively blocked by the densely-packed self-assembled monolayers of (3-Aminopropyl) triethoxysilane (APTES) on HfO_2_ effective layers.

**Figure 6:**
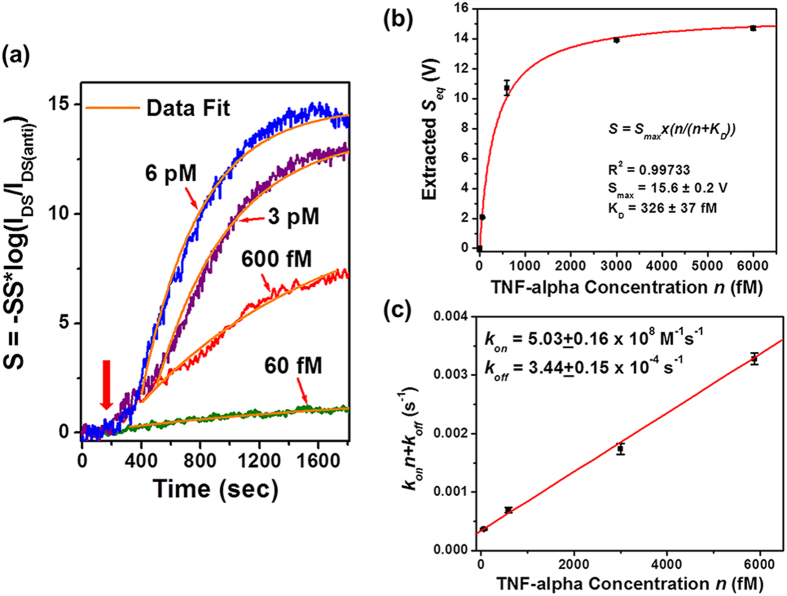
Time-dependent association kinetics of the antibody-(TNF-α) pair: (**a**) real-time sensor responses of antibody-(TNF-α) binding measured under different TNF-α concentrations (*n* = 60 fM, 600 fM, 3 pM, and 6 pM). Each of the response curves was measured from a different MoS_2_ transistor sensor and all responses were normalized using Equation (5). The rise parts of the binding response curves can be fitted with Equation (6). (**b**) The equilibrium-state responses (*S*_*eq*_) extracted from this fit plotted as a function of TNF-α concentration, which can be further fitted with Langmuir isotherm. The equilibrium constant (K_D_) is extracted to be 326 ± 37 fM. (**c**) The extracted *(k*_*on*_*n* + *k*_*off*_) data plotted as a function of TNF-α concentration (*n*). The linear fitting of this *(k*_*on*_*n* + *k*_*off*_*)-versus-n* graph results in rate constants of *k*_*on*_ = (5.03 ± 0.16) × 10^8^ M^−1^ s^−1^ and *k*_*off*_ = (3.44 ± 0.15) × 10^−4^ s^−1^.

The biosensor setup shown in Fig. 2(c) is used for measuring the time-dependent association/dissociation kinetics of the antibody-(TNF-α) pair. The details about microfluidic liquid handling and data recording are described in the Method and Material section. Figure 6(a) displays real-time sensor responses of antibody-(TNF-α) binding measured under different TNF-α concentrations (*i.e.*, *n* = 60 fM, 600 fM, 3 pM, and 6 pM). Each of the time-dependent response curves was measured from a different MoS_2_ transistor sensor and all as-measured *I*_*DS*_ responses were normalized using 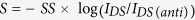 (*i.e.*, Equation (5) for calibrating subthreshold-regime responses). Figure S6 presents the detailed transfer characteristics of these transistor sensors measured before the input of TNF-α samples, from which the *SS* parameters required for normalizing *I*_*DS*_ responses were acquired. In addition, the operation points (*i.e.*, the fixed *V*_*G*_ and *V*_*DS*_ values, under which a real-time response curve was measured) are also labelled in Fig. S6. In Fig. 6(a), the red arrow indicates the onset time, at which the solutions with specific TNF-α concentrations were filled into the respective biosensors. The real-time response curves in Fig. 6(a) show that the association rate of the antibody-(TNF-α) pair increases with increasing TNF-α concentration. The rise segment of each real-time response curve can be well fitted with the first-order absorption equation (*i.e.*, Equation (6))^10^. In Equation (6), *S*_*eq*_ is the sensor response at the final equilibrium state; *k*_*on*_ and *k*_*off*_ are association and dissociation rates, respectively; *k*_*on*_*n* + *k*_*off*_ relates to the rising slope of the linear regime of the response curve. Table-1 lists the fitting results of *S*_*eq*_ and *(k*_*on*_*n* + *k*_*off*_) parameters for *n* = 60 fM, 600 fM, 3 pM, and 6 pM. These *S*_*eq*_ values extracted from the real-time binding responses are consistent with the sensor responses directly measured at the equilibrium state, that is, after a long incubation time of ~2 hours (*e.g.*, the equilibrium-state response data shown in Fig. 4(b)). In particular, Fig. 6(b) plots the extracted *S*_*eq*_ data as a function of TNF-α concentration, which can be also fitted with Langmuir isotherm. Here, the equilibrium constant (K_D_) is extracted to be 326 ± 37 fM and the maximum response (*S*_*max*_) is extracted to be 15.6 ± 0.2 V, which are consistent with those extracted from the equilibrium-state subthreshold-regime responses shown in Fig. 4(b).
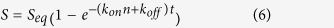


**Figure 7:**
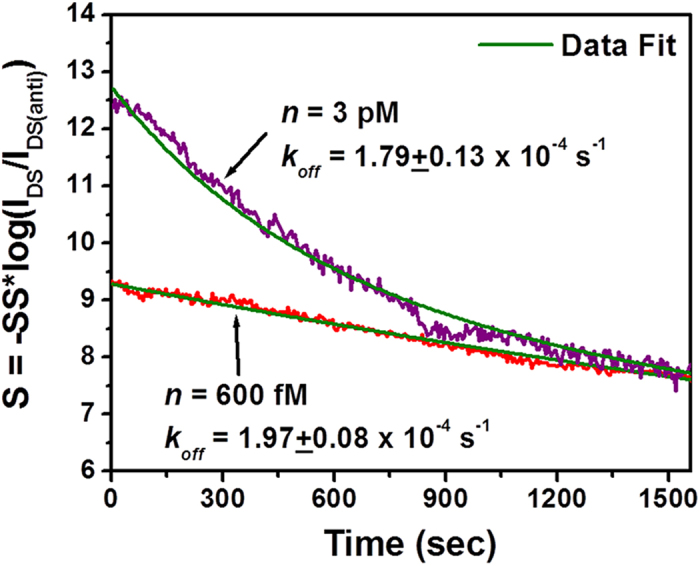
Time-dependent dissociation kinetics of the antibody-(TNF-α) pair measured from two MoS_2_ transistor sensors that were incubated in solutions with TNF-α concentrations of *n* = 600 fM and 3 pM for about 2 hours and subsequently rinsed with the pure buffer liquid flow.

To evaluate *k*_*on*_ and *k*_*off*_ parameters, the extracted *(k*_*on*_*n* + *k*_*off*_) data are plotted as a function of TNF-α concentration (*n*) (see Fig. 6(c)). The linear fitting results in rate constants of *k*_*on*_ = (5.03 ± 0.16) × 10^8^ M^−1^s^−1^ and *k*_*off*_ = (3.44 ± 0.15) × 10^−4^ s^−1^. It should be noted that this fit is not sensitive to the dissociation rate (*k*_*off*_) because of its small numerical value. To achieve a more precise quantification of *k*_*off*_, we directly measured the real-time dissociation kinetics of the antibody-(TNF-α) pair. Specifically, two as-functionalized MoS_2_ transistor biosensors were incubated in solutions with TNF-α concentration of 600 fM and 3 pM, respectively. The incubation time was more than 2 hours so that antibody-(TNF-α) association/dissociation processes reached to the equilibrium state. Afterwards, these fully incubated sensors were rinsed with pure buffer liquid flow and the calibrated sensor responses were recorded as a function of the lapsed time, as displayed in Fig. 7. Figure 7 shows that the sensor responses decreased with time, which was attributed to the unbinding events. The response curve measured from the device incubated with TNF-α concentration of 600 fM can be well fitted with a monoexponential decay function (*i.e.*, the desorption equation expressed in Equation (7)). In Equation (7), *S*_*r*_ represents the sensor response corresponding to the areal density of bound molecule residues after the desorption process. This fit results in *k*_*off*_ = (1.97 ± 0.08) × 10^−4^ s^−1^, from which the affinity equilibrium constant K_D_ can be also estimated to be K_D_=*k*_*off*_*/k*_*on*_= 392 fM. This K_D_ value is also consistent with those extracted from the equilibrium-state sensor responses (*i.e.*, K_D_ values extracted in Figs. 3 and 4). From this fit, *S*_*r*_ is extracted to be 3.0 ± 0.2 V and *S*_*eq*_ is 9.2 ± 0.4 V. This implies that ~30% of bound TNF-α molecules are expected to remain absorbed on the sensor even after a long rinsing process.
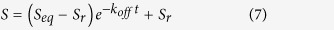

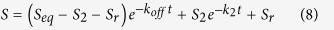


The response curve measured from the device incubated with TNF concentration of 3 pM can be hardly fitted with monoexponential Equation (7). We notice that it can be fitted with a bi-exponential decay equation (Equation (8)). This fit results in *S*_*eq*_ = 13.6 ± 1.0 V, *S*_*2*_ = 4.5 ± 0.2 V, *S*_*r*_ = 2.9 ± 0.3 V, *k*_*2*_ = (2.0 ± 0.16) × 10^−3^ s^−1^, and *k*_*off*_ = (1.79 ± 0.13) × 10^−4^ s^−1^. As reported by several previous works^10^,^55^,^56^, such a bi-exponential behavior of sensor responses is probably due to the multivalent antigen-antibody binding, which may become more prominent with increasing the analyte concentration. This explanation is reasonable because the antibody used in this work is polyclonal. To fully understand the association/dissociation kinetics of multivalent binding/unbinding processes, a more complicated model for describing antibody-(TNF-α) binding is needed.

Finally, it should be noted that for our current MoS_2_ transistor sensors, the calibrated sensor responses do not explicitly depend on HfO_2_ layer thickness (*t*_*HfO2*_). All sensors discussed above have 30 nm thick HfO_2_ effective layers. To further experimentally verify that the sensor responses of our sensors do not strongly depend on HfO_2_ layer thickness (*t*_*HfO2*_), we fabricated additional sensors with *t*_*HfO2*_ = 60 nm. Figure S7 (a) in the supplementary information displays the transfer characteristics of an exemplary sensor with *t*_*HfO2*_ = 60 nm, which were measured from a set of incremental TNF-α concentrations. From such transfer characteristics, we extracted calibrated subthreshold-regime responses (*S*) at *V*_*G*_ = −25 V (*V*_*T*_ ~ −10 V) and plotted them as a function of TNF-α concentration (*n*) (see the red stars shown in Fig. S7 (b)). This *S-n* relationship is consistent with those measured from the sensors with *t*_*HfO2*_ = 30 nm. This result proves that the calibrated sensor response values do not strongly depend on the HfO_2_ effective layer thickness.

## Conclusion

In conclusion, we presented important device physics and metrics for calibrating the responses of MoS_2_ transistor biosensors and demonstrated that multiple such sensors can be utilized to enable quantification of low-abundance biomarker molecules as well as the affinities and kinetics of antibody-mediated binding events. In particular, our biosensors exhibited a TNF-α detection limit at least as low as 60 fM. Such a low detection limit can be obtained in both linear and subthreshold regimes of MoS_2_ transistors. We further observed that the sensors operated in the subthreshold regime showed the higher current sensitivities in comparison with those in the linear regime. Such high subthreshold-regime sensitivities hold significant potential to further lower the TNF-α detection limit. In both transport regimes, the measured current signals can be normalized into response quantities independent of the transistor performance, which can effectively reduce the effect of sensor-to-sensor variation on biodetection results. Based on this calibration method, all sets of our biosensors can generate very consistent sensor responses with respect to TNF-α concentration and therefore a standard curve for TNF-α quantification. From this standard curve, the equilibrium constant of the antibody-(TNF-α) pair was extracted to be K_D_ = 369 ± 48 fM from linear-regime responses (or K_D_ = 424 ± 70 fM from subthreshold-regime responses). Furthermore, the real-time association/dissociation processes of the antibody-(TNF-α) pair were also quantified using multiple sensors. The association/dissociation rates were extracted to be *k*_*on*_ = (5.03 ± 0.16) × 10^8^ M^−1^s^−1^ and *k*_*off*_ = (1.97 ± 0.08) × 10^−4^ s^−1^, respectively. This work laid an important foundation for leveraging the excellent electronic properties of emerging atomically layered semiconductors in bio-assay applications as well as advanced the critical research capability in analyzing the biomolecule interactions with fM-level detection sensitivities. Notably, such capability would enable selection of antibodies with a high binding constant with respect to a specific target biomarker molecule, thereby providing a means to further improve the selectivity and fidelity of immunoassay.

## Materials and Methods

### Fabrication and Characterization of MoS_2_ transistor biosensors

The MoS_2_ transistors were fabricated using a microprinting method previously reported^57^. Few-layer-MoS_2_ channel thicknesses were specifically controlled to be 15–20 nm. Such a MoS_2_ thickness range has been demonstrated to result in the optimal field-effect mobility values for MoS_2_ transistors^52^,^53^. The transistor channel lengths (*L*) were ~5 μm and the channel widths (*W*) ranged from 5 to 8 μm. Ti (5 nm)/Au (50 nm) electrode pairs served as drain (D) and source (S) contacts, which were created using photolithography followed with metal deposition and lift-off. The p^+^-Si substrates were used as the back gates (G). Thermally grown SiO_2_ layers (300 nm thick) were used as the back-gate dielectrics. Such 300 nm thick SiO_2_ layers can enable a simple color coding method for us to quickly identify MoS_2_ flakes with suitable thicknesses (*i.e.*, 15–20 nm)^58^. All electrical measurements were performed using an HP-4145B semiconductor parameter analyzer.

### Bio-functionalization of MoS_2_ transistor biosensors

Figure S1 in the supporting information illustrates the protocol for functionalizing the HfO_2_ effective layer of a MoS2 transistor sensor with anti-human TNF-α antibody receptors for detecting TNF-α molecules. First, an as-fabricated transistor biosensor is immersed in 5% (3-Aminopropyl) triethoxysilane (APTES, purchased from Sigma-Aldrich Co. LLC.) in ethanol for 1 hour. After the incubation, the sensor is rinsed with phosphate buffered saline (PBS) and blown dry by nitrogen gas. After this step, the HfO_2_ effective layer is silanized with an APTES monolayer. The device is subsequently immersed in 5% gluteraldehyde (GA) (purchased from Sigma-Aldrich Co. LLC.) in PBS for 2 hours followed by rinsing with PBS. Afterwards, anti-human TNF-α antibody (from eBioscience, Inc.) of 50 ug/ml concentration in DI water is dropped on the sensor and incubated for 1 hour. For studying the equilibrium-state sensor responses, the as-functionalized sensor is incubated with TNF-α solutions with incremental concentrations (*i.e.*, *n* = 60 fM, 300 fM, 600 fM, 3 pM, and 6 pM; the incubation time for each of the concentrations: ~2 hours). The incubation is performed using the setup illustrated in Fig. 1(d). After each incubation process, the transfer characteristics of the transistor sensor are measured.

### Quantification of the time-dependent association/dissociation kinetics of the antibody-(TNF-α) pair

An as-functionalized MoS_2_ transistor biosensor is covered with a polydimethylsiloxane (PDMS) block bearing a microfluidic channel (10 mm in length, 200 μm in width, 50μm in height), as illustrated in Fig. 1(e). A motorized syringe pump is used for driving the analyte flows into and out of the microfluidic channel through an inlet/outlet tubing kit (tube diameter: 0.75 mm). At the beginning of the measurement of a real-time sensor response curve associated with antibody-(TNF-α) binding, deionized (DI) water is injected into the sensor with flow rate of 5 μL/min. At the same time, the MoS_2_ transistor is biased under a given set of *V*_*G*_ and *V*_*DS*_. After the *I*_*DS*_ value is stabilized, the analyte solution with a specific TNF-α concentration is injected into the sensor.

## Additional Information

**How to cite this article**: Nam, H. *et al.* Multiple MoS_2_ Transistors for Sensing Molecule Interaction Kinetics. *Sci. Rep.* **5**, 10546; doi: 10.1038/srep10546 (2015).

## Supplementary Material

Supporting Information

## Figures and Tables

**Table 1:** The fitting results of the real-time sensor response curves shown in Fig. 5(a) that are fitted with Equation (6). The table lists the extracted *S*_*eq*_ and *(k*_*on*_ *n* + *k*_*off*_) parameters for *n* = 60 fM, 600 fM, 3 pM, and 6 pM.

	**n=60 *fM***	**600 *fM***	**3 *pM***	**6 *pM***
*S*_*eq*_ (V)	2.07 ± 0.03	10.7 ± 0.5	13.9 ± 0.06	14.7 ± 0.13
(*k*_*on*_*n*±*k*_*off*_) (s^−1^)	(3.68 ± 0.15)× 10^−4^	(6.94 ± 0.46)×10^−4^	(1.74 ± 0.10)×10^−3^	(3.28 ± 0.10)×10^−3^
