# Supplementary material for: Multiple MoS2 Transistors for Sensing Molecule Interaction Kinetics
Source: Sci Rep. 2015 May 27;5:10546. doi: 10.1038/srep10546 (PMC4444958; doi:10.1038/srep10546)
Supplement: Supporting Information [file srep10546-s1.pdf]

Multiple MoS_2 Transistors for Sensing Molecule Interaction Kinetics

Hongsuk Nam[1, †], Bo-Ram Oh[1, †], Pengyu Chen[1], Mikai Chen[1], Sungjin Wi[1], Wenjie Wan[2], Katsuo Kurabayashi[1], and Xiaogan Liang[,1]*

[1] Department of Mechanical Engineering, University of Michigan, Ann Arbor, MI 48109

[2] University of Michigan-Shanghai Jiao Tong University Joint Institute and Department of Physics and Astronomy, Shanghai Jiao Tong University, Shanghai 200240, China

*Contact Email: xiaoganl@umich.edu

[†] These authors contributed equally

Supporting Information

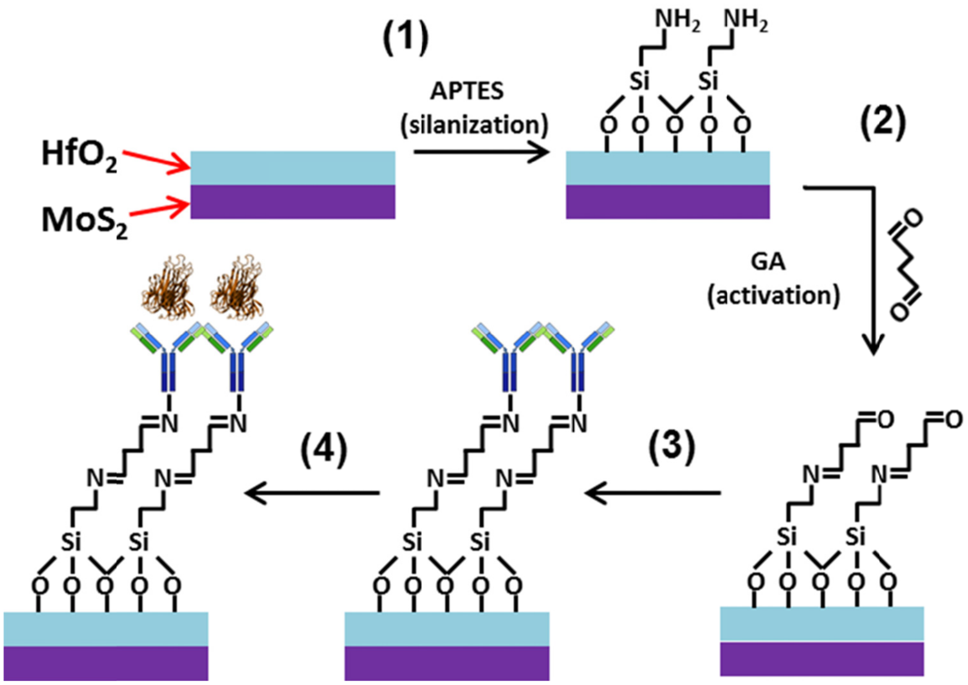

Figure S1. Protocol for functionalizing a MoS_2 transistor sensor with anti-human TNF-α antibody receptors for detecting TNF-α molecules: (1) Immerse the HfO_2-coated MoS_2 transistor sensor into a 5% APTES solution and incubate for 1 hour. (2) The HfO_2 surface silanized with APTES reacts with a 5% solution of glutaraldehyde (GA) in phosphate buffered saline (PBS) for 2 hours, followed by rinsing with PBS buffer. (3) The HfO_2 surface is then incubated with an anti-human TNF-α antibody solution for 1 hour. (4) The as-functionalized sensor is incubated with TNF-α solutions with incremental concentrations (2 hours for each concentration) for studying the sensor responses at the equilibrium state and the affinity of the antibody-(TNF-α) pair, or the device is subjected to a TNF-α flow in a microfluidic channel for quantifying the time-dependent association/dissociation kinetics of the antibody-(TNF-α) pair.

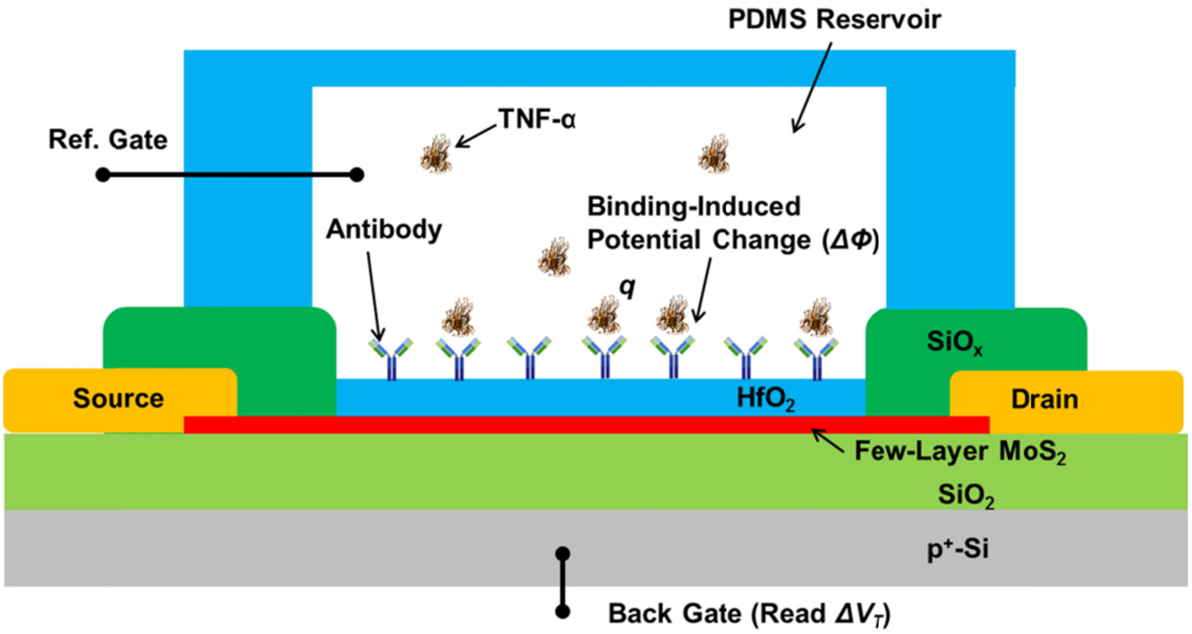

Figure S2. The dual-gate thin-film transistor biosensor model: The binding of TNF-α molecules with the antibody receptors functionalized on the HfO_2 effective layer can cause a potential change ($\Delta\Phi$) on this effective layer. $\Delta\Phi$ can be evaluated using $\Delta\Phi = \dfrac{qN_{TNF}}{C_{HfO2}}$, where q is the effective charge brought to the HfO_2 effective layer through a single antibody-(TNF-α) binding event (here, q is the effective charge sensed by the transistor, and the screening effect due to the electrical double layer in solvent has been involved into q); N_{TNF} is the total number of TNF-α molecules bound to the HfO_2 effective layer; C_{HfO2} is the total capacitance of the HfO_2 effective layer. This $\Delta\Phi$ induces a change in the conductive charge ($\Delta Q=C_{HfO2}\Delta\Phi$) in the MoS_2 channel. This ΔQ can cause a shift of the threshold voltage (ΔV_T) measured from the back gate (note: not measured from the top gate), and ΔV_T can be evaluated by $\Delta V_T=\Delta Q/C_{SiO2}=(C_{HfO2}/C_{SiO2})\Delta\Phi= \dfrac{qN_{TNF}}{C_{SiO2}}$, where C_{SiO2} is the capacitance of the back-gate dielectric layer. Furthermore, N_{TNF} can be calculated using $N_{TNF} = \sigma_{TNF} A$, where σ_{TNF} is the areal density of bound TNF-α molecules on

the effective layer and A is the total sensor area. C_{SiO2} can be calculated using C_{SiO2} = $k_{SiO2}\varepsilon_0 A/d_{SiO2}$, where d_{SiO2} and k_{SiO2} are the thickness and dielectric constant of the SiO_2 back-gate dielectric layer, respectively; ε_0 is the vacuum permittivity. Therefore, $\Delta V_T = \dfrac{qN_{TNF}}{C_{SifO2}} = \dfrac{qd_{SiO2}\sigma_{TNF}}{k_{SiO2}\varepsilon_0}$.

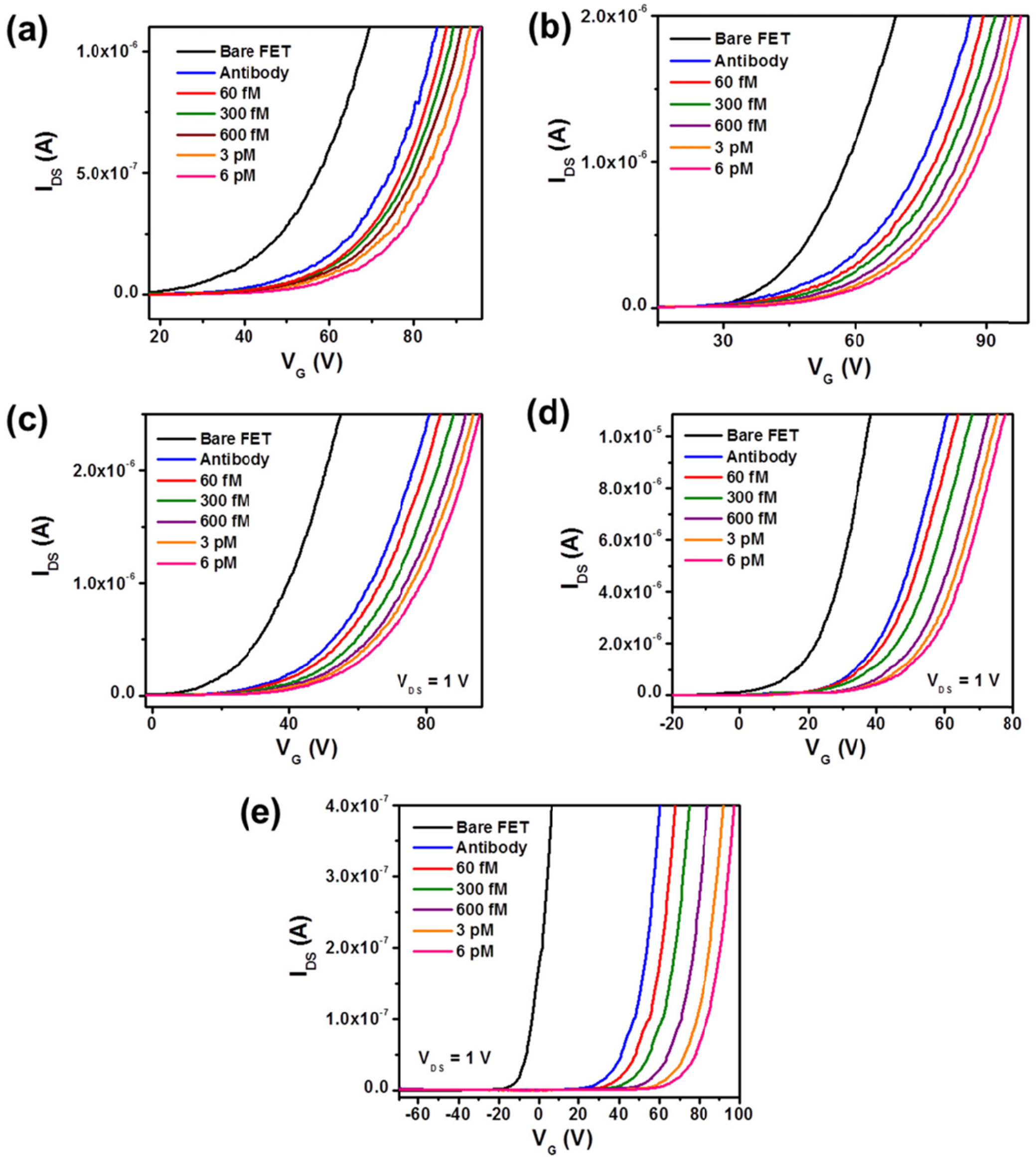

Figure S3. Linear-regime sensor responses at the equilibrium state: The transfer characteristics of five different MoS_2 transistor sensors measured at various biodetection stages, following the sequence of (1) bare transistor, (2) antibody functionalization, and inputs of TNF-α solutions with concentrations of (3) 60 fM, (4) 300 fM, (5) 600 fM, (6) 3 pM, and (7) 6 pM. The calibrated linear-regime sensor responses from these five devices are plotted in Fig. 3 (b) with respect to TNF-α concentration.

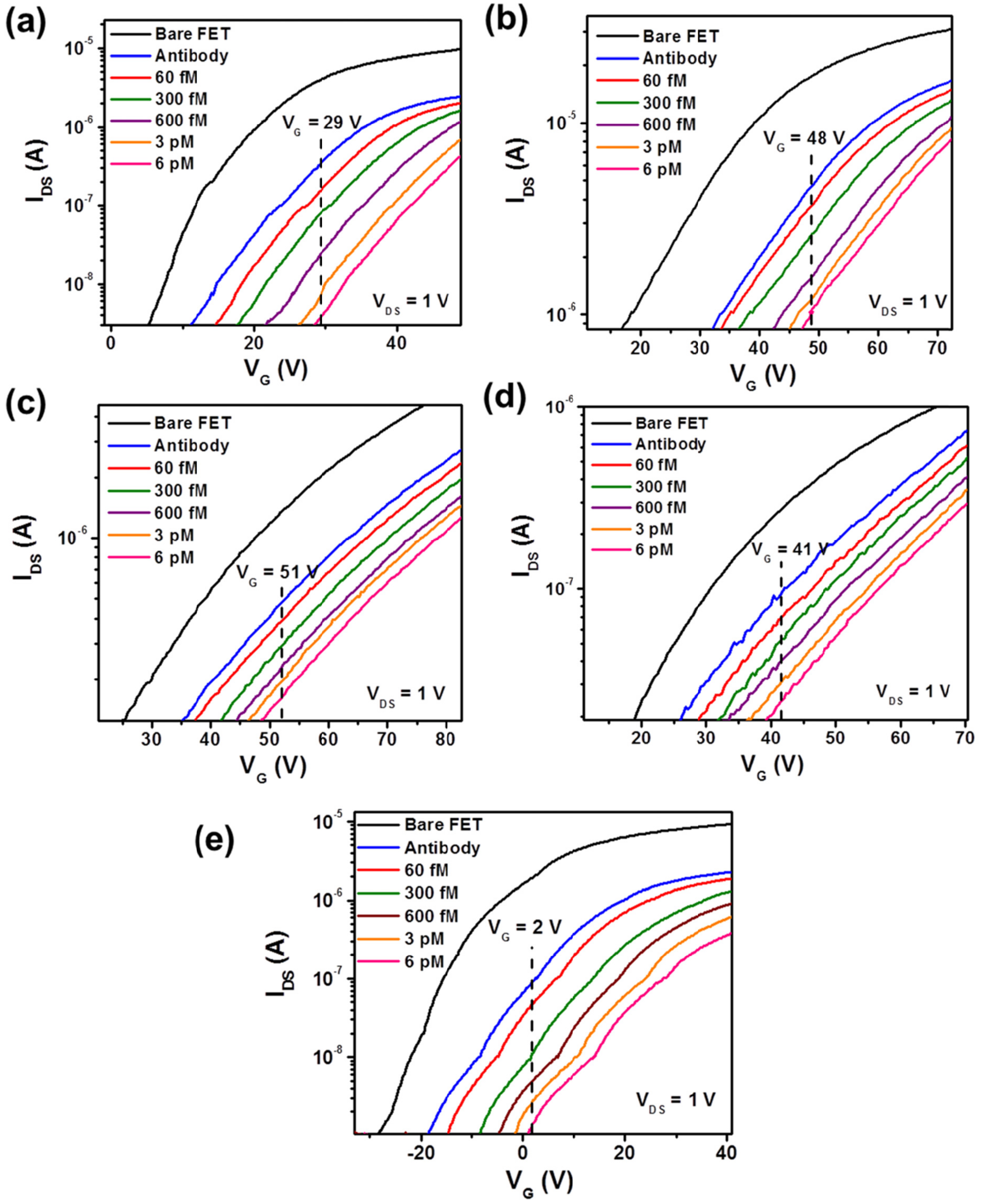

Figure S4. Subthreshold-regime sensor responses at the equilibrium state: The transfer characteristics of five different MoS_2 transistor sensors measured at various biodetection stages,

following the sequence of (1) bare transistor, (2) antibody functionalization, and inputs of TNF-α solutions with concentrations of (3) 60 fM, (4) 300 fM, (5) 600 fM, (6) 3 pM, and (7) 6 pM. The calibrated subthreshold-regime sensor responses from these five devices are plotted in Fig. 4 (b) with respect to TNF-α concentration.

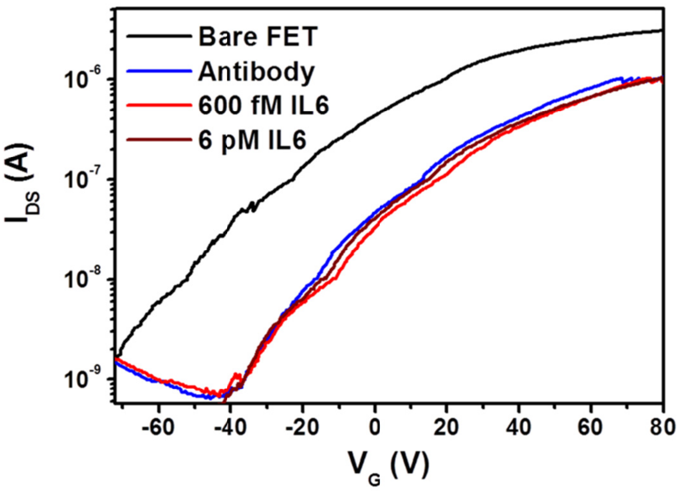

Figure S5. A negative control test of the detection specificity of MoS_2 transistor biosensors: The transfer characteristics of a control sensor measured at stages of (1) bare transistor, (2) antibody functionalization (still functionalized with anti-human TNF-α antibody receptors), and inputs of IL-6 solutions with concentrations of (3) 600 fM and (4) 6 pM.

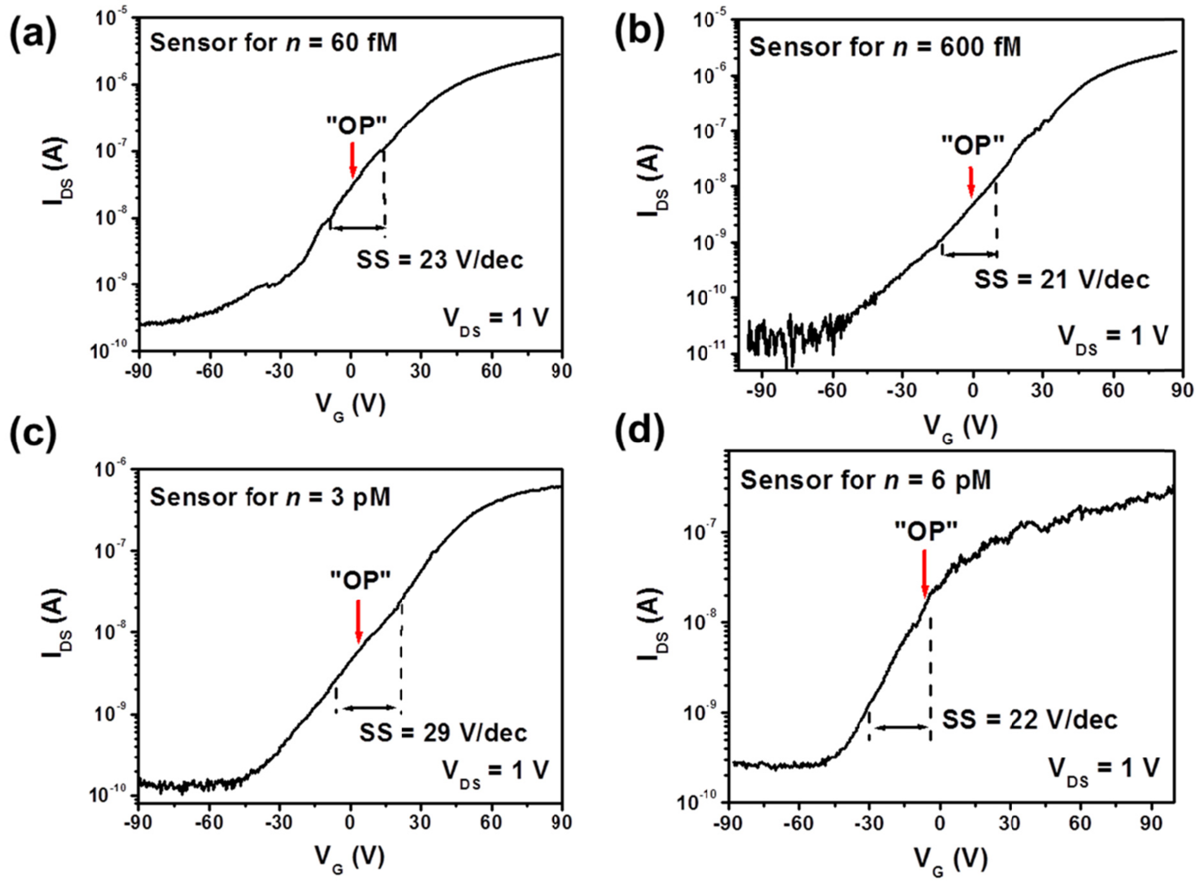

Figure S6. Transfer characteristics of four different MoS_2 transistor biosensors measured before the input of TNF-α samples, from which the subthreshold-swing (*SS*) parameters were acquired for normalizing the real-time subthreshold-regime sensor responses (Equation (5)). These sensors were utilized to quantify the real-time kinetics of antibody-(TNF-α) binding under different TNF-α concentrations (n) of (a) 60 fM, (b) 600 fM, (c) 3 pM, and (d) 6 pM. The operation points (OP, *i.e.*, the fixed V_G and V_{DS} values, under which a real-time response curve was measured) are also labelled by the red arrows.

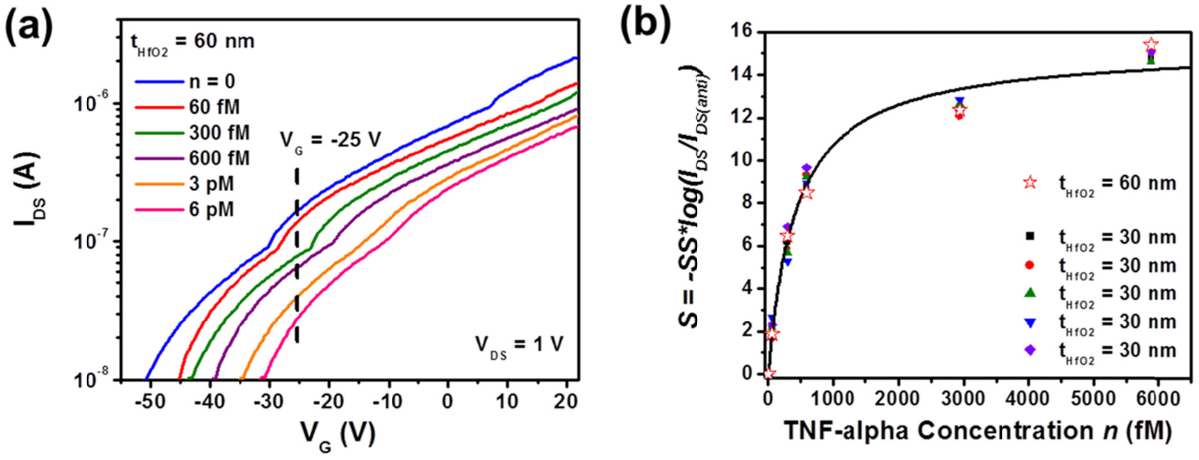

Figure S7. Sensor responses measured in the subthreshold regime of a MoS_2 transistor biosensor with a 60 nm thick HfO_2 effective layer (*i.e.*, t_{HfO2} = 60 nm): (a) transfer characteristics of the MoS_2 transistor sensor with t_{HfO2} = 60 nm, which were measured from a set of incremental TNF-α concentrations (*i.e.*, n = 0, 60 fM, 300 fM, 600 fM, 3 pM, and 6 pM; (b) The calibrated subthreshold-regime responses (*S*) measured from this sensor (labelled as red stars) with respect to TNF-α concentration (*n*). This *S*-*n* relationship measured from this sensor with t_{HfO2}=60 nm is consistent with those measured from the sensors with t_{HfO2} = 30 nm. This result proves that the calibrated sensor response values do not strongly depend on the HfO_2 effective layer thickness.
